# Draft Genome Sequence of *Photobacterium halotolerans* S2753, Producer of Bioactive Secondary Metabolites

**DOI:** 10.1128/genomeA.00535-14

**Published:** 2014-06-12

**Authors:** Henrique Machado, Maria Månsson, Lone Gram

**Affiliations:** a Department of Systems Biology, Technical University of Denmark, Kgs. Lyngby, Denmark; b Novo Nordisk Foundation Center for Biosustainability, Technical University of Denmark, Hørsholm, Denmark

## Abstract

We report here the whole draft genome sequence of marine isolate *Photobacterium halotolerans* S2753, which produces the known antibiotic holomycin and also ngercheumicins and solonamides A and B, which interfere with virulence of methicillin-resistant *Staphylococcus aureus* strains by interacting with the quorum-sensing system.

## GENOME ANNOUNCEMENT

*Photobacterium halotolerans* strain S2753 is a Gram-negative marine bacterium that was isolated from a mussel during the Danish global expedition Galathea 3 (1). It is able to inhibit the growth of both Gram-positive and Gram-negative pathogens such as *Staphylococcus aureus* (human pathogen) and *Vibrio anguillarum* (fish pathogen) (1, 2). This specific strain has been shown to produce a variety of secondary metabolites with distinct bioactivities (3, 4). It produces holomycin, a known antibiotic (2), as well as a series of ngercheumicin analogues (5) (K. Adachi, Y. Kawabata, H. Kasai, M. Katsuta, and Y. Shizuri, 13 September 2007, Japanese patent application JP 2007–230911 A) and most interestingly two novel cyclodepsipeptides named solonamide A and solonamide B (3, 4).

Antimicrobial resistance in pathogens is a serious problem derived both from the rapid adaptation of microorganisms to antimicrobials and the recent stagnancy in antibiotic discovery. *S. aureus* is a versatile pathogen that has caused particular concern recently due to fast antimicrobial resistance development among both clinical and community isolates (6–8). Therefore, therapeutics that do not exert a killing effect, but rather interfere with virulence of the organism, e.g., by interfering with the quorum-sensing systems, have been studied and developed in recent years (9). *P. halotolerans* S2753 produces compounds capable of reducing the expression of virulence in *S. aureus* (10). Both the ngercheumicins and solonamides interfere with the Agr system of *S. aureus*, with solonamide B having the strongest interaction (10). Also, this compound can compete with the four classes of autoinducing peptides (AIPs) in *S. aureus*, although interference with AIP class III is weak.

This announcement reports the draft genome sequence of *P. halotolerans* S2753, a strain producing chemical compounds with interesting features for application in biotechnological and medical fields.

High-purity genomic DNA was extracted by successive phenol:chloroform:isoamyl-alcohol purification steps followed by precipitation with isopropanol, treatment with RNase, and final purification and precipitation steps (11). Quantification was done in 1% agarose gel electrophoresis using a NanoDrop spectrometer (Saveen Werner, Sweden) and Qubit 2.0 analyzer (Invitrogen, United Kingdom).

The sequencing of the genomes was performed by the Beijing Genomic Institute (Shenzhen, China). Libraries of 500 bp were used for 100-bp paired-end sequencing of genomes using Illumina sequencing technology on an HiSeq2000 with a minimum coverage of 100. This generated 5.03 million reads of clean data, making a total of 462,754,934 bases. The data were assembled into 82 contigs (>200 bp) covering 4,533,138 bases by *de novo* assembly in CLC Genomic Workbench, version 7 (CLC Bio, Aarhus, Denmark).

In order to predict clusters involved in secondary metabolite synthesis, the reported genome was run on the analysis pipeline antiSMASH (12,13), revealing the potential of this specific strain. Besides the holomycin cluster, 11 other putative clusters encoding secondary metabolites were identified in the *P. halotolerans* S2753 genome.

### Nucleotide sequence accession numbers.

This whole-genome shotgun project has been deposited at DDBJ/EMBL/GenBank under the accession no. JMIB00000000. The version described in this paper is version JMIB01000000.

## References

[1] Gram L Melchiorsen J Bruhn JB 2010 Antibacterial activity of marine culturable bacteria collected from a global sampling of ocean surface waters and surface swabs of marine organisms. Mar. Biotechnol. 12:439–451. 10.1007/s10126-009-9233-y 19823914

[2] Wietz M Mansson M Gotfredsen CH Larsen TO Gram L 2010 Antibacterial compounds from marine *Vibrionaceae* isolated on a global expedition. Mar. Drugs 8:2946–2960. 10.3390/md8122946 21339958 PMC3039463

[3] Mansson M Gram L Larsen TO 2011 Production of bioactive secondary metabolites by marine *Vibrionaceae*. Mar. Drugs 9:1440–1468. 10.3390/md9091440 22131950 PMC3225927

[4] Mansson M Nielsen A Kjærulff L Gotfredsen CH Wietz M Ingmer H Gram L Larsen TO 2011 Inhibition of virulence gene expression in *Staphylococcus aureus* by novel depsipeptides from a marine photobacterium. Mar. Drugs 9:2537–2552. 10.3390/md9122537 22363239 PMC3280567

[5] Kjaerulff L Nielsen A Mansson M Gram L Larsen TO Ingmer H Gotfredsen CH 2013 Identification of four new agr quorum sensing-interfering cyclodepsipeptides from a marine photobacterium. Mar. Drugs 11:5051–5062. 10.3390/md11125051 24351904 PMC3877902

[6] Herold BC Immergluck LC Maranan MC Lauderdale DS Gaskin RE Boyle-vavra S Leitch CD Daum RS 1998 *Staphylococcus aureus* in children with no identified predisposing. Risk 279:593–598 10.1001/jama.279.8.593 9486753

[7] Diep BA Gill SR Chang RF Phan TH Chen JH Davidson MG Lin F Lin J Carleton HA Mongodin EF Sensabaugh GF Perdreau-Remington F 2006 Complete genome sequence of USA300, an epidemic clone of community-acquired meticillin-resistant *Staphylococcus aureus*. Lancet 367:731–739. 10.1016/S0140-6736(06)68231-7 16517273

[8] Lowy FD 2007 Secrets of a superbug. Nat. Med. 13:1418–1420. 10.1038/nm1207-1418 18064034

[9] Shoham M 2011 Antivirulence agents against MRSA. Future Med Chem. 3:775–777. 10.4155/fmc.11.43 21644821

[10] Nielsen A Månsson M Bojer MS Gram L Larsen TO Novick RP Frees D Frøkiær H Ingmer H 2014 Solonamide B inhibits quorum sensing and reduces *Staphylococcus aureus* mediated killing of human neutrophils. PLoS One 9:e84992. 10.1371/journal.pone.0084992 24416329 PMC3885660

[11] Sambrook J Russel DW 2001 Molecular cloning: A laboratory manual. Cold Spring Harbor Laboratory Press, Cold Spring Harbor, New York

[12] Medema MH Blin K Cimermancic P de Jager V Zakrzewski P Fischbach MA Weber T Takano E Breitling R 2011 antiSMASH: rapid identification, annotation and analysis of secondary metabolite biosynthesis gene clusters in bacterial and fungal genome sequences. Nucleic Acids Res. 39:W339–W346. 10.1093/nar/gkr466 21672958 PMC3125804

[13] Blin K Medema MH Kazempour D Fischbach MA Breitling R Takano E Weber T 2013 antiSMASH 2.0—a versatile platform for genome mining of secondary metabolite producers. Nucleic Acids Res. 41:W204–W212. 10.1093/nar/gkt449 23737449 PMC3692088

